# Regulation of Skeletal Muscle Oxidative Capacity and Muscle Mass by SIRT3

**DOI:** 10.1371/journal.pone.0085636

**Published:** 2014-01-15

**Authors:** Ligen Lin, Keyun Chen, Waed Abdel Khalek, Jack Lee Ward, Henry Yang, Béatrice Chabi, Chantal Wrutniak-Cabello, Qiang Tong

**Affiliations:** 1 USDA/ARS Children’s Nutrition Research Center, Department of Pediatrics, Baylor College of Medicine, Houston, Texas, United States of America; 2 INRA, UMR 866-Dynamique Musculaire et Métabolisme, Montpellier, France; 3 Department of Medicine and Department of Molecular Physiology & Biophysics, Baylor College of Medicine, Houston, Texas, United States of America; University of Texas Health Science Center at San Antonio, United States of America

## Abstract

We have previously reported that the expression of mitochondrial deacetylase SIRT3 is high in the slow oxidative muscle and that the expression of muscle SIRT3 level is increased by dietary restriction or exercise training. To explore the function of SIRT3 in skeletal muscle, we report here the establishment of a transgenic mouse model with muscle-specific expression of the murine SIRT3 short isoform (SIRT3M3). Calorimetry study revealed that the transgenic mice had increased energy expenditure and lower respiratory exchange rate (RER), indicating a shift towards lipid oxidation for fuel usage, compared to control mice. The transgenic mice exhibited better exercise performance on treadmills, running 45% further than control animals. Moreover, the transgenic mice displayed higher proportion of slow oxidative muscle fibers, with increased muscle AMPK activation and PPARδ expression, both of which are known regulators promoting type I muscle fiber specification. Surprisingly, transgenic expression of SIRT3M3 reduced muscle mass up to 30%, likely through an up-regulation of FOXO1 transcription factor and its downstream atrophy gene MuRF-1. In summary, these results suggest that SIRT3 regulates the formation of oxidative muscle fiber, improves muscle metabolic function, and reduces muscle mass, changes that mimic the effects of caloric restriction.

## Introduction

Caloric restriction (CR) prolongs animal lifespan and delays the onset of age-related diseases [1], [2]. In skeletal muscle, CR increases insulin sensitivity, modulates protein turnover and protects against the aging-related decline of mitochondrial activity and muscle function [3]–[5]. However, the molecular mechanism underlying the effects of CR in skeletal muscle is poorly understood.

The sirtuin family of genes has been proposed as possible mediators for the effects of CR. SIRT3, a sirtuin family member of NAD^+^-dependent deacetylase, is the major mitochondrial protein deacetylase [6]. SIRT3 expression is increased in response to fasting or caloric restriction [7]–[9]. SIRT3 deacetylates many mitochondrial enzymes to orchestrate metabolic alteration. For example, in the liver, SIRT3 deacetylates long-chain acyl CoA dehydrogenase (VCAD) to boost fatty acids β-oxidation [10], 3-hydroxy-3-methylglutaryl CoA synthase 2 (HMGCS2) to increase ketogenesis [11], acetyl-CoA synthetase 2 (ACS2) to utilize acetate [12], [13], and ornithine transcarbamoylase (OTC) to detoxificate urea [9]. SIRT3 also regulates mitochondrial electron transporta chain, such as complex I subunit NDUFA9 [14], complex II succinate dehydrogenase [15], and ATP synthase ATP5A [16]. Furthermore, SIRT3 deacetylates MnSOD [17]–[19] and isocitrate dehydrogenase 2 [20] to augment anti-oxidant action. SIRT3-deficient mice have greatly decreased levels of tissue adenosine triphosphate (ATP) [14], impaired cold tolerance when fasted [10], and more susceptibility to cardiac hypertrophy [21], [22], breast cancer [23] and high-fat diet-induced metabolic syndrome [24], [25].

The function of SIRT3 in skeletal muscle is not fully characterized. We have reported previously that caloric-restricted mice have increased SIRT3 expression in both white and brown adipose tissue [7] and skeletal muscle [8]. We also found that the oxidative soleus muscle has higher SIRT3 expression than does glycolytic extensor digitorum longus or gastrocnemius muscles and that expression of SIRT3 in skeletal muscle is elevated by fasting and exercise training [8]. Human studies revealed that muscle SIRT3 expression is down-regulated with age and up-regulated by endurance training [26]. Conversely, sedentary elder human subjects have reduced muscle expression of SIRT3 and the peroxisome proliferator-activated receptor gamma coactivator-1α (PGC-1α), compared to young and active elder controls [27]. SIRT3 deficiency leads to decreased muscle oxidative capacity and oxidative stress, resulting in defects of muscle insulin signaling [24].

AMPK is a ubiquitous heterotrimeric serine/threonine protein kinase, which functions as a fuel sensor in many tissues, including skeletal muscle [28]. Activated AMPK stimulates ATP-generating catabolic pathways, such as fatty acid uptake and β-oxidation by phosphorylating and inactivating acetyl-CoA carboxylase (ACC) [28]. In addition, AMPK activation represses ATP-consuming processes, such as lipogenesis, protein synthesis and other biosynthetic pathways [28], [29]. Activation of AMPK promotes a switch to type I fibers and increased exercise capacity [30], [31]. In the muscle of SIRT3 knockout mice, we observed a down-regulation of AMPK phosphorylation [8].

Forkhead transcription factors are key components of the insulin/IGF signaling cascade, a conserved pathway regulating metabolism and aging [32]. Nutrient deprivation, such as calorie restriction or fasting, elevates muscle expression of forkhead transcription factors FOXO1 and FOXO3 [33], [34], which activate the expression of the E3 ubiquitin ligases atrogin-1/MAFbx and MuRF-1 to promote muscle protein degradation [35]. Overexpression of FOXO1 in skeletal muscle can cause muscle atrophy [36], [37]. On the other hand, activation of the atrophy genes and programs may have beneficial effects by accelerating protein turnover and clearance of damaged or aggregated proteins that could otherwise compromise muscle health [38]. For example, increased expression of FOXO and its downstream target 4EBP in *Drosophila* muscle extended lifespan and protected against aging-associated muscle protein aggregation and loss of muscle strength [39].

To date, no study of a gain-of-function mouse model of SIRT3 in the skeletal muscle has been reported. The murine SIRT3 gene expresses three different protein isoforms, the long isoforms SIRT3M1 and SIRT3M2 and the short isoform SIRT3M3, with variable mitochondrial localization efficiency and protein stability [40]–[44]. Different transcription variants of the same gene may play distinct roles. For example, the PGC-1α4 transcript has a specific function not shared by the regular PGC-1α1 transcript [45]. So far, only one SIRT3 transgenic mouse model has been reported, in which cardiac expression of the short-form SIRT3 protects mice againist angiotensin II-induced or isoproterenol-induced cardiac hypertrophy and fibrosis [21]. Therefore, we generated transgenic mice with skeletal muscle-specific expression of SIRT3M3-FLAG to investigate the function of SIRT3M3 in skeletal muscle. We uncovered the role of SIRT3 in driving the formation of oxidative type I muscle fibers and in causing a reduction of muscle mass.

## Materials and Methods

### Generating Transgenic Mice

The mouse SIRT3M3 (short form) coding sequence with FLAG tag in pCR blunt II-TOPO vector was described previously [7]. The 0.8 kb SIRT3-FLAG fragment was cut with XhoI and HindIII and then inserted into pBluescript II k/s vector with human growth hormone (hGH) polyadenylation sequence at XhoI and HindIII sites. The 6.5 kb promoter of muscle creatine kinase (MCK) was cut with XhoI from the pMCK6.5-pUC118 plasmid (kindly provided by S. D. Hauschka) [46] and then cloned into XhoI at the 5′ of the SIRT3. The pBS-MCK-SIRT3-FLAG-hGH plasmid was digested with BssHII, and the 7.9 kb transgene construct was injected into fertilized C57BL/6 mouse oocytes by the Genetically Engineered Mouse Core at Baylor College of Medicine, Houston, Texas. Multiple transgenic lines were established and two lines were analyzed and reported here. Wild-type (WT) and skeletal muscle-specific SIRT3 transgenic mice (MCK-SIRT3M3) were housed under controlled temperature and lighting (75±1°F; 12h light-dark cycle) with free access to food and water. Mice were rested for at least one week between each test and more than two weeks before being euthanized for tissue collection. All experiments were approved by the Animal Care Research Committee of the Baylor College of Medicine.

### Real-time PCR

Total RNA of muscle and heart was isolated using TRIzol Reagent (Invitrogen, Carlsbad, CA, USA) following the manufacturer’s instructions. The cDNA was synthesized using the SuperScript III First-Strand Synthesis System for real-time polymerase chain reaction (RT-PCR) (Life Technologies, Carlsbad, CA, USA). RT-PCR was performed on a LightCycler using the FastStart DNA Master SYBR Green (Roche Diagnostics, Indianapolis, IN, USA) according to the protocol provided by the manufacturer. The expression level of 18S ribosomal RNA was used as a control for qPCR. Sequences of primers used for RT-PCR were as follows: SIRT3-F 5′-CGGCTCTATACACAGAACATCGA-3′ and SIRT3-R 5′-GTGGGCTTCAACCAGCTTTG-3′; atrogin-1-F 5′-GCAAACACTGCCACATTCTCTC-3′ and atrogin-1-R 5′-CTTGAGGGGAAAGTGAGACG-3′; MuRF-1-F 5′-ACCTGCTGGTGGAAAACATC-3′ and MuRF-1-R 5′-CTTCGTCCTTGCACATC-3′; 18S ribosomal RNA-F 5′-AACGAGACTCTGGCATGCTAACTAG-3′ and 18S ribosomal RNA-R 5′-CGCCACTTGTCCCTCTAAGAA-3′.

### Indirect Calorimetry

Indirect calorimetry was conducted in a computer-controlled, open-circuit system (Oxymax System) that was part of an integrated Comprehensive Lab Animal Monitoring System (CLAMS; Columbus Instruments, Columbus, OH, USA). Calorimetry and daily food intake data were acquired during the 3 days of the experimental period, after a 3-day acclimation period. Oxygen consumption (VO_2_) and carbon dioxide production (VCO_2_) were measured for each chamber every 40 minutes and calculated by Oxymax software (v. 5.9). Energy expenditure was calculated as EE = 3.815×VO_2_+1.232×VCO_2_. A photobeam-based activity monitoring system detected and recorded ambulatory movements, including rearing and climbing movements, in every cage. The sensors for detection of movements operated efficiently in both light and dark phases, allowing continuous recording. Total activity was defined as the combination of horizontal (*x* level) and vertical (*z* level, rearing) activity.

### Glucose Tolerance Test

Following overnight withdrawal of food, mice were administrated glucose (2 g/kg) by intraperitoneal injection glucose tolerance test (IP-GTT) or oral gavage GTT (OGTT), and blood samples for determination of glucose were collected from the tail vein at the indicated times. Glycemia was measured using OneTouch Ultra2 glucometer (Lifescan, Milpitas, CA, USA).

### Treadmill Endurance Capacity Test

A 15° uphill treadmill protocol was performed using an Exer-3/6 open treadmill (Columbus Instruments, Columbus, OH, USA) according to guidelines from the American Physiological Society [47]. Mice were run first at 6 meter per minute. The treadmill speed then was increased by 2 meter per minute every 2 minutes until mice were exhausted. Nudging was used during the treadmill exercise to help mice stay on the track. Exhaustion was defined as spending more than 10 seconds without attempting to reenter the treadmill. Maximum exercise capacity was estimated from each run-to-exhaustion trial using three parameters: the duration of the run (in minutes), the distance run (in meters), and the vertical work performed (in g⋅m) [48]. VO_2_ and VCO_2_ also were recorded during the treadmill exercise.

### Inverted Grid Hanging Test

Fatigability of limbs was tested using the inverted-grid hanging test. Mice were placed individually on the center of an invertible 40×40 cm wire grid, mounted 0.5 meter above a padded surface. After the grid was gently inverted, the time was recorded for which the mouse was able to hang on, to a maximum of 5 minutes. Each mouse was tested three times with 30-minute intervals. The average hanging times were calculated for each mouse.

### String Test

A string test was conducted in three consecutive trials (separated by 10-minute intervals). Each mouse was suspended by its fore paws on a horizontally stretched wire, and the time the mouse took to catch the wire with its hind paws was recorded. If the mouse was unable to climb on after one minute, the trial was stopped.

### Histological Analyses

Skeletal muscle tissues (quadriceps and gastrocnemius) were fixed overnight in 10% formalin at room temperature, dehydrated, and embedded in paraffin. Then, tissue blocks were sectioned at 5 µm for hematoxylin and eosin (H&E) staining. For ATPase staining, skeletal muscle samples were frozen in optimal cutting temperature (OCT) compound in liquid nitrogen-cooled isopentane, and transverse 10 µm sections were prepared and stained using the metachromatic myosin ATPase staining method as described [49]. The type I fibers, type IIa fibers, and type IIb/IIx fibers were stained to deep blue, white blue-violet, and light blue, respectively.

### Hybridoma Cells Culture

Monoclonal antibodies directed against adult myosin heavy chain (MHC) isoforms were harvested from hybridoma cell lines (American Type Culture Collection, Manassas, VA, USA): BA-D5 (IgG and anti-MHC I), SC-71 (IgG and anti-MHC IIa), and BF-F3 (IgM and anti-MHC IIb), following product instructions [50], [51]. Briefly, cells were cultured in Complete Growth Medium (Dulbecco’s Modified Eagle’s Medium supplied with 4 mM L glutamine, 4.5 g/l glucose, 1.5 g/l sodium bicarbonate, and 10% fetal bovine serum) until confluence occurred and then were switched to fresh medium. After 6 days, supernatant was harvested and used for Western blot analysis or frozen and stored in −80°C freezer.

### Mitochondrial Isolation

Before dissection was undertaken, mice were housed in normal conditions. After mice were weighed and euthanized by isoflurane, cervical dislocation was performed. Gastrocnemius, quadriceps, and plantaris muscles were quickly excised and immediately placed into ice-cold buffer (100 mM KCl, 5 mM MgSO_4_, 5 mM EDTA, 50 mM Tris-HCl, pH = 7.4). Mitochondria were fractionated by differential centrifugation as described previously [52], [53]. Briefly, muscles were freed of connective tissues, minced, homogenized with an Ultra-turax homogenizer, and treated with Subtilisin A (0.1 mg/g wet muscle). Mitochondria were separated by centrifugation at 8000 *g*, then at 800 *g*. Finally, mitochondria were pelleted from the supernatant at 9000 *g*. Mitochondria were re-suspended in 100 mM KCl, 10 mM MOPS, pH 7.4. Mitochondrial protein content was determined using the Bradford assay [54], and the yield was expressed as mg of mitochondrial proteins per gram of muscle wet weight.

### Mitochondrial Respiration

Mitochondria respiration was measured using the high-resolution Oxygraph-2k (OROBOROS Instruments, Innsbruck, Austria). Mitochondria were incubated in two sealed chambers (37°C) containing 2 ml of MIRO5 respiration medium (0.5 mM EGTA, 3 mM MgCl_2_.6H_2_O, 65 mM KCl, 20 mM taurine, 10 mM KH_2_PO_4_, 20 mM HEPES, 110 mM sucrose and 1 g/l BSA, pH 7.1) [55]. Resting rate (state 4) was evaluated in the presence of 2.5 mM malate, 5 mM glutamate, and 5 mM succinate; ADP-stimulated rate (state 3) was determined after addition of 0.5 mM ADP. The integrity of the mitochondria was checked using NADH addition during state-3 measurement. The increase in respiration was less than 10% and not significantly different between WT and transgenic mice, demonstrating that mitochondria were fully functional. Data acquisition and analysis were performed using Oxygraph-2k-DatLab software version 4.3 (OROBOROS Instruments, Innsbruck, Austria). The respiratory control ratio (RCR) was set as the ratio of oxygen consumption at state 3 over oxygen consumption at state 4.

### Western Blot Analysis

Skeletal muscle tissues were lysed in lysis buffer (50 mM Tris, 50 mM KCl, 20 mM NaF, 1 mM Na_3_VO_4_, 10 mM EDTA, 1% NP-40, 1 mM PMSF, 5 µg/ml leupeptin, pH 8.0). Sarcoplasmic and nuclear proteins of the quadriceps were extracted using NE-PER Nuclear and Cytoplasmic Extraction Reagents kit (Thermo Fisher Scientific, Inc., Rockford, IL, USA). Protein concentration was determined with BCA protein assay kit (Thermo Fisher Scientific). Twenty micrograms of protein from each sample was separated by SDS-PAGE and electro-transferred to nitrocellulose membrane for immunoblot analysis. The following antibodies were used: anti-SIRT3 (1∶1000) that we have previously developed [8], anti-FLAG (Sigma, St. Louis, MO, USA; F1804, 1∶2000), anti-α-tubulin (Sigma; T5168, 1∶100,000), anti-β-actin (Santa Cruz Biotechnology, Santa Cruz, CA, USA; sc-1616, 1∶1000), anti-phospho-AMPKα (Thr172) (Cell Signaling Technology, Danvers, MA, USA; 2535, 1∶1000), anti-AMPKα (Cell Signaling Technology; 2532, 1∶1000), anti-phospho-acetyl CoA carboxylase (Ser79) (Millipore Corporation, Billerica, MA, USA; 05–673, 1∶1000), anti-acetyl CoA carboxylase (Millipore Corporation; 07–439, 1∶1000), anti-PPARγ (Santa Cruz Biotechnology; sc-7273, 1∶1000), anti-PPARδ (Santa Cruz Biotechnology; sc-1987, 1∶1000), anti-phospho-FOXO-1 (Santa Cruz Biotechnology; sc-19808, 1∶1000), anti-FOXO-1 (Santa Cruz Biotechnology; sc-67140, 1∶1000), HRP-conjugated anti-mouse (Bio-Rad, Richmond, CA, USA; 170–6516, 1∶30,000), anti-rabbit (Bio-Rad; 170–6515, 1∶30,000). The SuperSignal West Pico Chemiluminescent kit (Thermo Scientific**)** was used as substrates.

For the detection of the SIRT3M3-FLAG transgene protein, SignalBoost Immunoreaction Enhancer Kit (Millipore Corporation) was used together with the primary and secondary antibodies. The SuperSignal® West Femto Maximum Sensitivity Substrate kit (Pierce) was also used.

### Statistics

The data are represented as the mean ± standard error. Statistical significance was determined using the two-tail Student’s t-test to compare each transgenic line of mice against littermate wild type controls. For energy expenditure of mice, we used Minitab to perform ANCOVA analysis. *P*<0.05 was considered to be statistically significant.

## Results

### Generation of Muscle-specific SIRT3 Transgenic Mice

**Figure 1:**
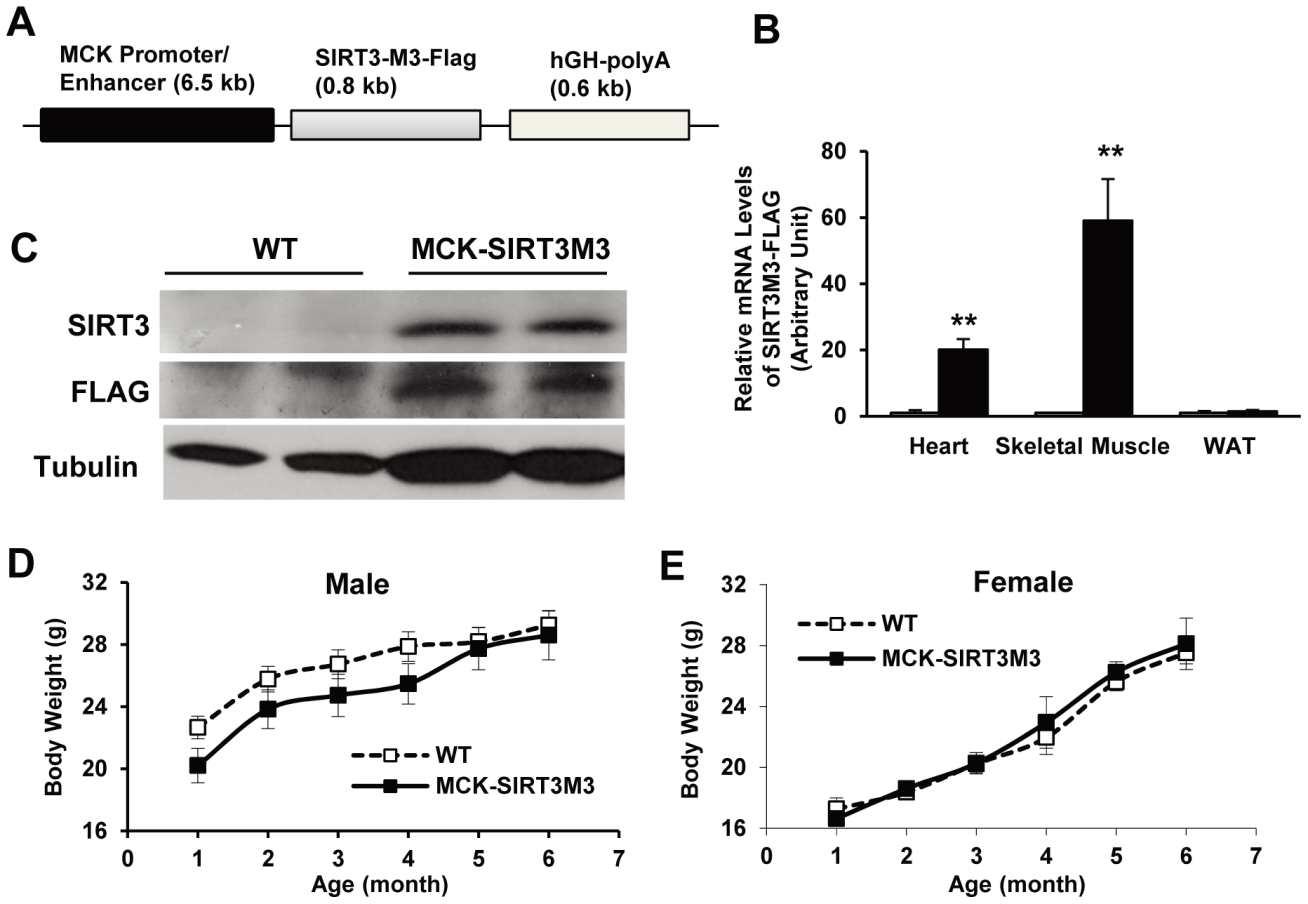
Creation of muscle-specific SIRT3 transgenic mice. (A): Diagram of the transgene construct. The SIRT3-M3-FLAG transgene was under the control of the 6.5kb muscle creatine kinase (MCK) promoter/enhancer with the human growth hormone polyadenylation site at the 3′ end. (B): The mRNA expression of the SIRT3-M3-FLAG transgene in heart, quadriceps muscle and white adipose tissue, was measured using real-time RT-PCR. The results were normalized with cyclophilin expression and presented as relative to WT controls. *n* = 5. (C): The SIRT3M3-FLAG transgene product was detected in the quadriceps muscle lysates by Western blot analysis using anti-SIRT3 or anti-FLAG antibodies. (D): Body weight of male WT and MCK-SIRT3M3 mice. *n* = 6–9. (E): Body weight of female WT and MCK-SIRT3M3 mice. *n* = 5–9. **P<0.01 between WT and MCK-SIRT3M3 mice.

SIRT3 expression is high in slow oxidative muscle and is increased by exercise training or caloric restriction [8]. To mimic exercise or nutrient deprivation-stimulated SIRT3 expression and directly assess the role of SIRT3 in skeletal muscle, we generated transgenic mice with C-terminal FLAG-tagged murine SIRT3M3 cDNA [43] under the control of the promoter/enhancer element of MCK [56] (Fig. 1A). We have established multiple transgenic lines and performed detailed analysis of one line (data presented in the Figures of main text) and confirmed some key findings in a second line (data presented in the Supporting Figures) to rule out the positional effect of transgene integration. In agreement with previous characterization of the MCK promoter/enhancer element [57], the transgene mRNA was highly expressed in skeletal muscle, with a lower expression in heart and no expression in other tissues, such as adipose tissue (Fig. 1B). However, the protein product of SIRT3M3-FLAG transgene was expressed at a low level, and we were able to detect it only by using a high-sensitivity Western blot method (Fig. 1C). This is not entirely surprising, as we have reported that, compared to the long SIRT3 M1 and M2 isoforms, the short SIRT3M3 protein has a short half-life and is quickly degraded by ubiquitination and the proteasome system [44].

### MCK-SIRT3M3 Mice had Increased Oxygen Consumption and Lipid Utilization

**Figure 2:**
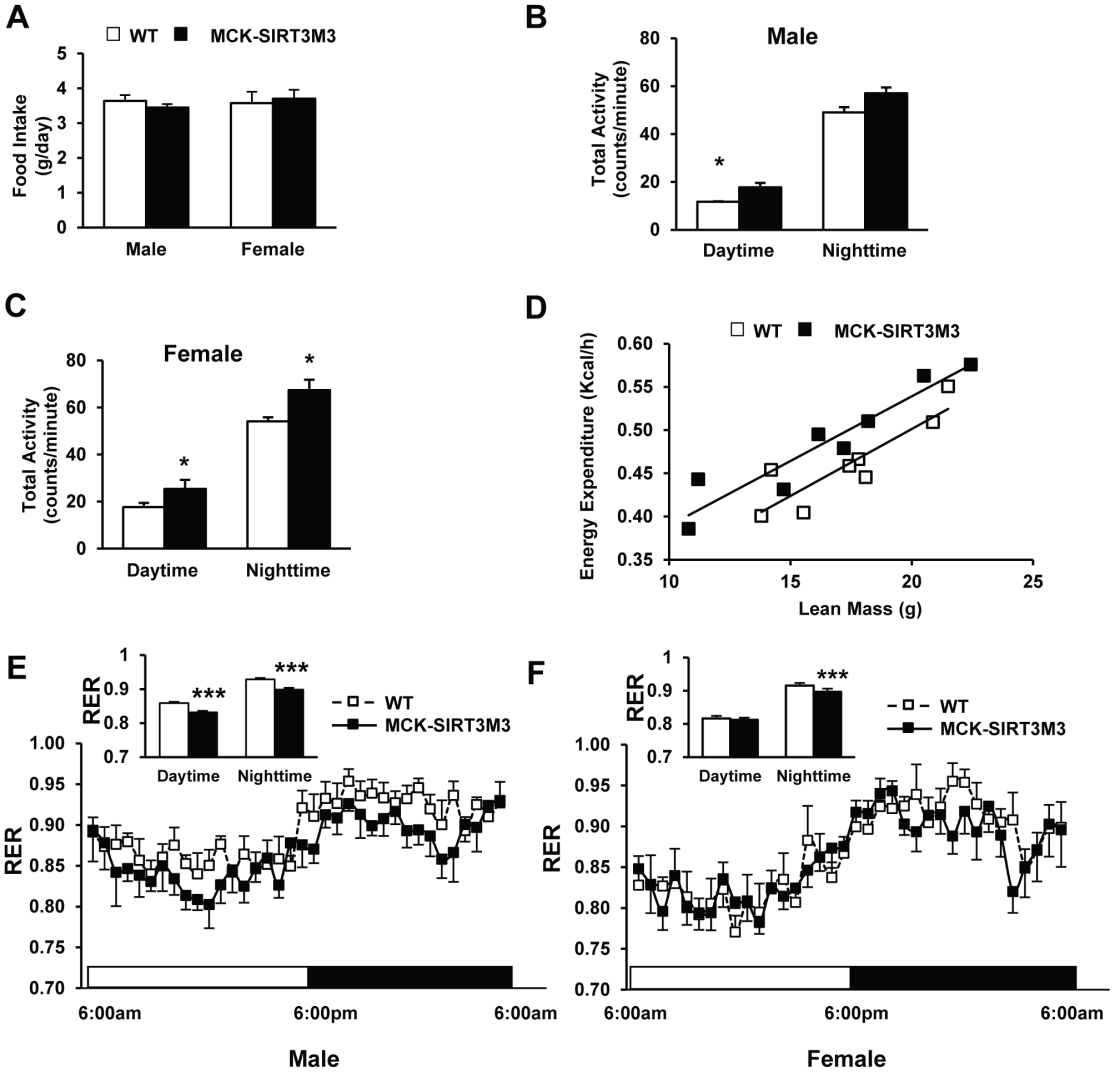
Metabolic characterization of MCK-SIRT3M3 transgenic mice. (A): Daily food intake of 6-month old WT and MCK-SIRT3M3 mice. (B): Total locomotor activity at daytime and nighttime of 6-month old male WT and MCK-SIRT3M3 mice. (C): Total locomotor activity at daytime and nighttime of 6-month old female WT and MCK-SIRT3M3 mice. (D): Correlation of energy expenditure and lean body mass, for female WT and MCK-SIRT3M3 mice. (E and F): Respiratory exchange rate (RER) of WT and MCK-SIRT3M3 mice. *n* = 6. *P<0.05, ***P<0.001 between WT and MCK-SIRT3M3 mice.

When fed with regular chow diet, neither male nor female transgenic mice exhibited body weight differences (Fig. 1D, 1E, S1A, and S1B). Next, we used indirect calorimetry to characterize the metabolic profiles of MCK-SIRT3M3 mice. The daily food intake of WT and MCK-SIRT3M3 mice was comparable for both males and females (Fig. 2A and S1C). However, the spontaneous physical activity of MCK-SIRT3M3 mice was increased for both males and females in one transgenic line (Fig. 2B and 2C) but not the other (Fig. S1D). In addition, both male and female MCK-SIRT3M3 mice had similar VO_2_ and VCO_2,_ with or without normalization by body weight (data not shown). When examining energy expenditure as a function of lean body mass, we found that the female transgenic mice and their WT littermates lay on two separate regression lines parallel to each other (Fig. 2D), but the male mice did not (data not shown). We performed analysis of covariance using lean body mass as a covariate [58]. The results showed that energy expenditure after adjustment of the differences of lean body mass was 8.7% higher for the female transgenic mice (p = 0.006) but was not different for the male transgenic mice (p = 0.67). Interestingly, both male and female transgenic mice had lower respiratory exchange rate (RER) (Fig. 2E, 2F, and S1E), indicating that the MCK-SIRT3M3 mice favor lipid utilization as a fuel source. Taken together, the indirect calorimeter study revealed that the MCK-SIRT3M3 mice had higher energy expenditures and lower respiratory quotients, without significant alteration of food intake. As muscle is the major tissue for glucose disposal, we also performed GTT to evaluate glucose homeostasis of the transgenic mice. Both IP-GTT (Fig. S2) and OGTT (data not shown) revealed no significant difference in glucose homeostasis.

### SIRT3M3 Enhanced Muscle Oxidative Capacity but Reduced Muscle Strength and Motor Coordination

**Figure 3:**
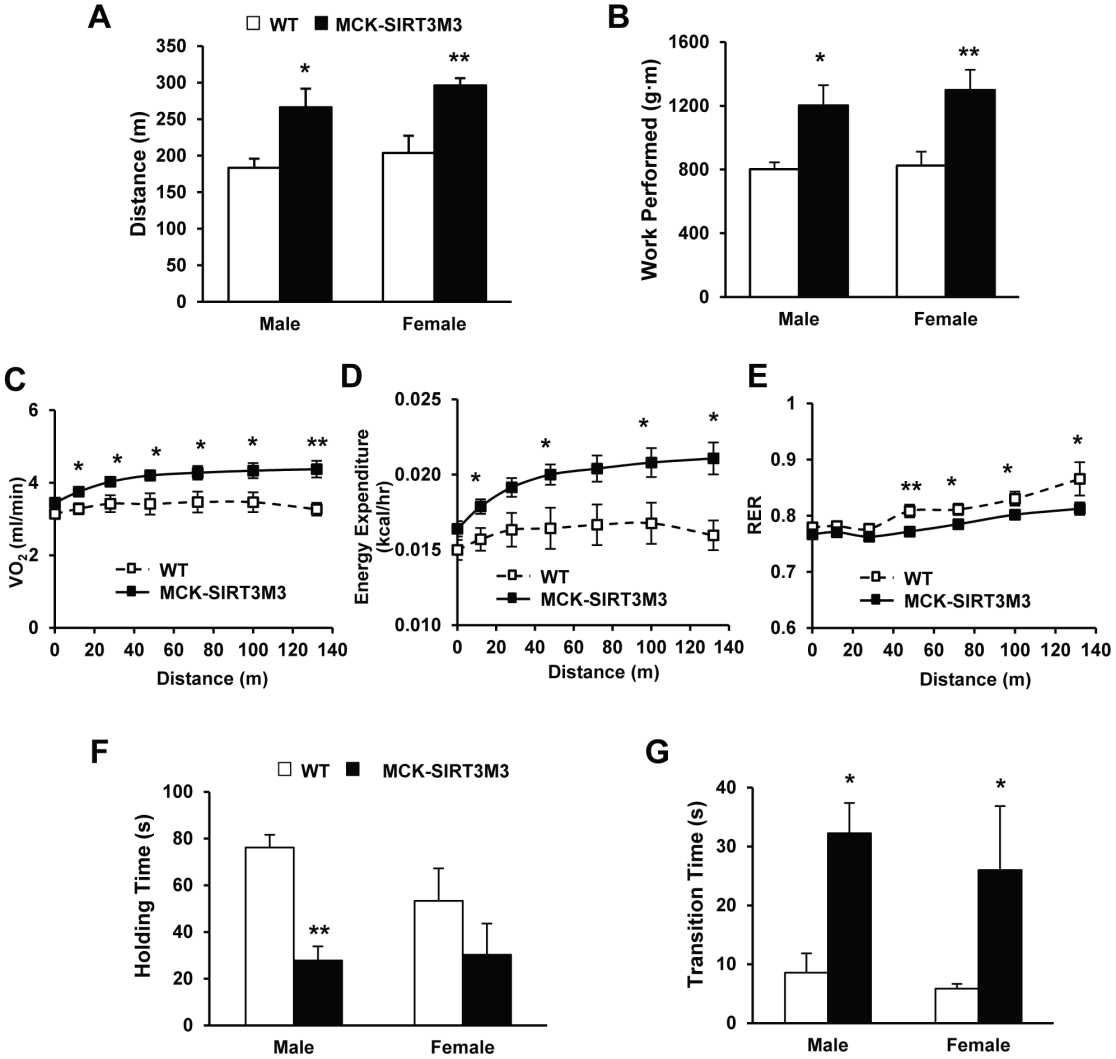
Oxidative capacity and muscle strength of MCK-SIRT3M3 transgenic mice. (A): Running distance of WT and MCK-SIRT3M3 mice on treadmill. (B): Work performed of WT and MCK-SIRT3M3 mice on treadmill. (C, D and E): Oxygen consumption, heat production, and respiratory exchange rate (RER) of male WT and MCK-SIRT3M3 mice during treadmill. *n* = 5–7. (F): Holding time of WT and MCK-SIRT3M3 mice on inverted grid mesh test. (G): Transition time of WT and MCK-SIRT3M3 mice climbing on string test. *n* = 6–10. *P<0.05, **P<0.01 between WT and MCK-SIRT3M3 mice.

We next examined the exercise performances of these mice, when they were forced to run to exhaustion on an up-hill treadmill. Exhaustion closely follows the attainment of the anaerobic threshold, which in turn closely correlates with oxidative capacity [59]. We found the male MCK-SIRT3M3 mice were exhausted after an average time of 18.5±1.0 minutes, compared to 14.7±0.6 minutes for WT littermates. The female MCK-SIRT3M3 mice were exhausted after an average time of 19.8±0.4 minutes, compared to 15.6±1.1 minutes for WT littermates. In terms of running distances, that of the MCK-SIRT3M3 mice was 45% further than that of WT animals (Fig. 3A and S3A), indicating that MCK-SIRT3M3 mice performed significantly more work than did WT controls, calculated as 1201.9 joules versus 802.1 joules for male and 1298.6 joules versus 824.6 joules for female, respectively (Fig. 3B). During treadmill exercises, MCK-SIRT3M3 mice consumed more oxygen, produced more heat, and had lower RER (Fig. 3C, 3D and 3E). Hence, specific expression of SIRT3M3 in skeletal muscle is sufficient to increase the oxidative capacity and exercise performance, with a preference of the utilization of fatty acids as energy source.

When we investigated the muscle strength of the transgenic mice using an inverted grid hanging test and a string test, the WT mice hung on the inverted grid mesh 2.7-fold (male) and 1.8-fold (female) longer than did the MCK-SIRT3M3 mice (Fig. 3F and S3B). Similarly, when hanging by forelimbs on a string wire, WT mice took less time to climb up than MCK-SIRT3M3 mice did (Fig. 3G and S3C). These results suggest that expression of SIRT3M3 in skeleton muscle reduces muscle strength.

### Transgenic Expression of SIRT3M3 Augmented Type I Muscle Fiber Formation

**Figure 4:**
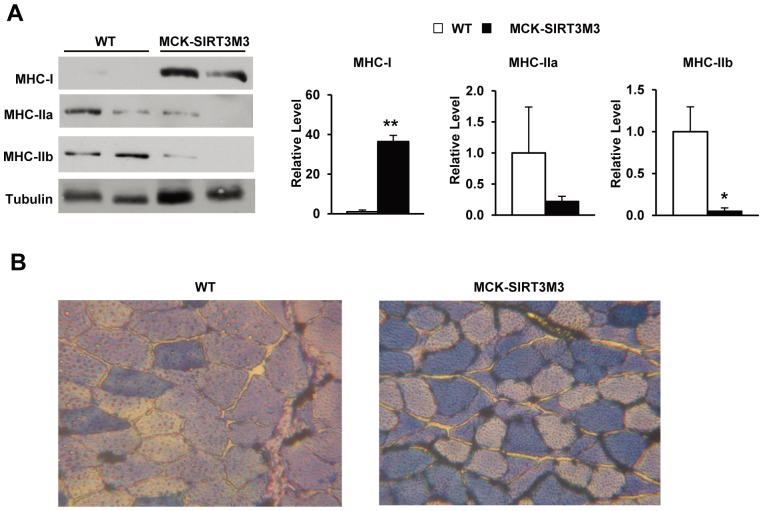
Fiber type characterization of muscle from MCK-SIRT3M3 transgenic mice. (A): Protein levels of three different myosin heavy chains (MHC) in quadriceps muscle, evaluating by Western blotting. The relative levels of MHC proteins were normalized by tubulin levels. (B): ATPase staining of quadriceps muscle.

Endurance exercise relies largely on oxidative fibers in skeletal muscle. The enhancement of the capacity for aerobic exercise of MCK-SIRT3M3 mice suggested these mice might have an increase of oxidative muscle fibers. The Western blot analysis performed on muscle extracts using antibodies specific for the MHC-I, IIa and IIb isotypes to evaluate the composition of fiber in MCK-SIRT3M3 mice revealed a marked induction of MHC-I protein and suppression of MHC-IIa and IIb proteins in quadriceps muscle of MCK-SIRT3M3 mice (Fig. 4A). Metachromatic myosin ATPase staining performed to examine the muscle fiber type distribution revealed a substantial amount of type I muscle fibers in the quadriceps of the MCK-SIRT3M3 mice (Fig. 4B).

**Figure 5:**
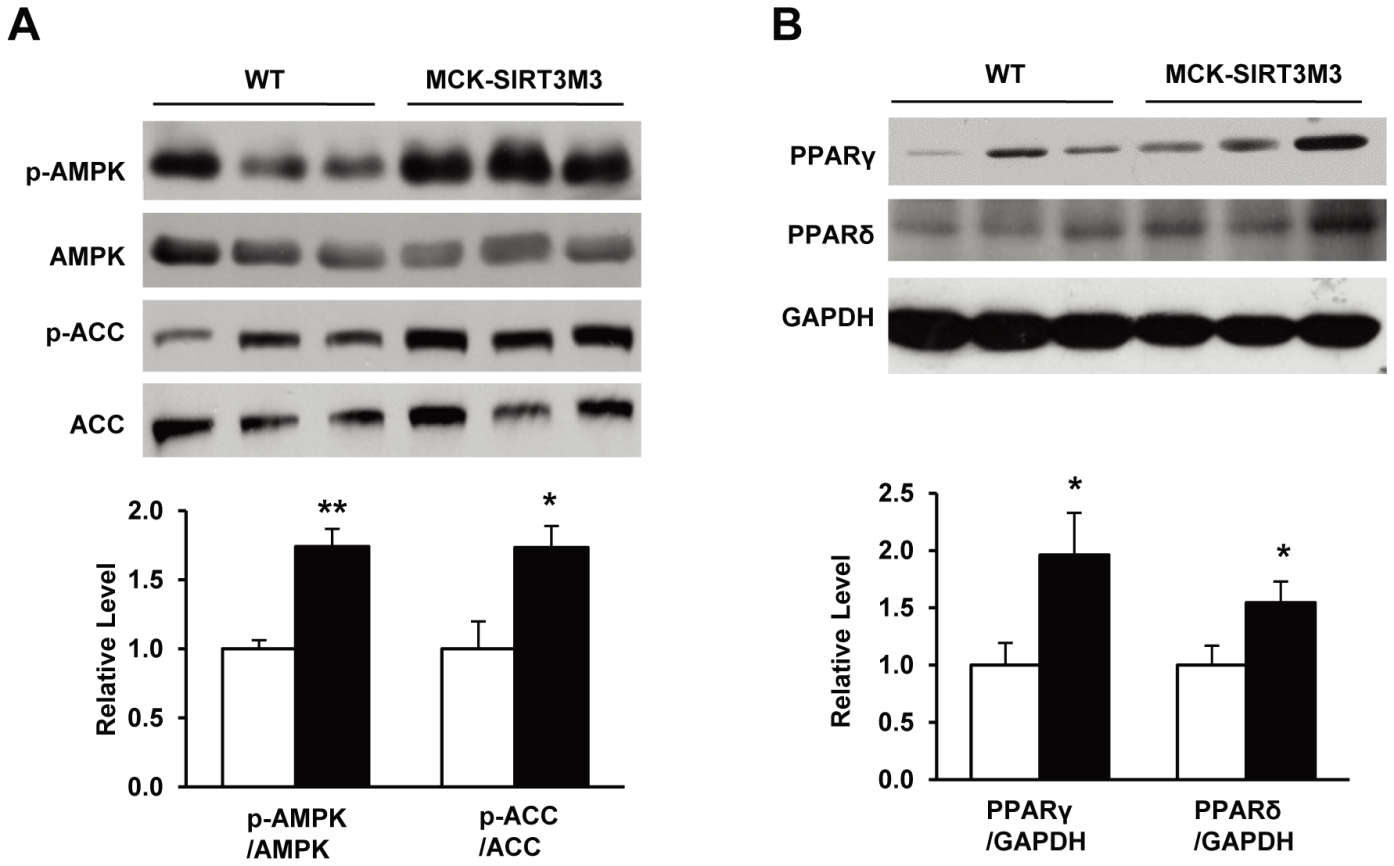
The analysis of proteins responsible for muscle fiber determination in MCK-SIRT3M3 transgenic mice. (A): Phosphorylation of AMPK and ACC in quadriceps muscle of WT and MCK-SIRT3M3 mice. (B): Protein levels of PPARγ and PPARδ in quadriceps muscle of WT and MCK-SIRT3M3 mice.

Since activation of AMPK promotes a switch to type I fibers and increased exercise capacity [30], [31], and we observed a down-regulation of AMPK phosphorylation in the muscle of SIRT3 knockout mice [8], we examined the AMPK phosphorylation in the muscles of the transgenic mice. We found that the MCK-SIRT3M3 mice had significantly elevated levels of phosphorylated AMPK without any change in the total AMPK protein levels (Fig. 5A). Furthermore, the level of phosphorylated ACC, a substrate of AMPK, was also increased in MCK-SIRT3M3 mice (Fig. 5A). PPARδ has been shown to induce a shift toward slow oxidative type I fiber type [48]. Interestingly, Western blot analysis revealed that PPARγ and PPARδ expression were increased in the quadriceps of MCK-SIRT3M3 mice (Fig. 5B). The increase of PPARδ was also detected in EDL muscle (data not shown). However, PGC-1α and PGC-1β protein levels were not changed in the muscle of the transgenic mice (data not shown). Taken together, overexpression SIRT3 in skeletal muscle drives the switch of muscle fiber types, mainly through the activation of AMPK and PPARδ.

**Figure 6:**
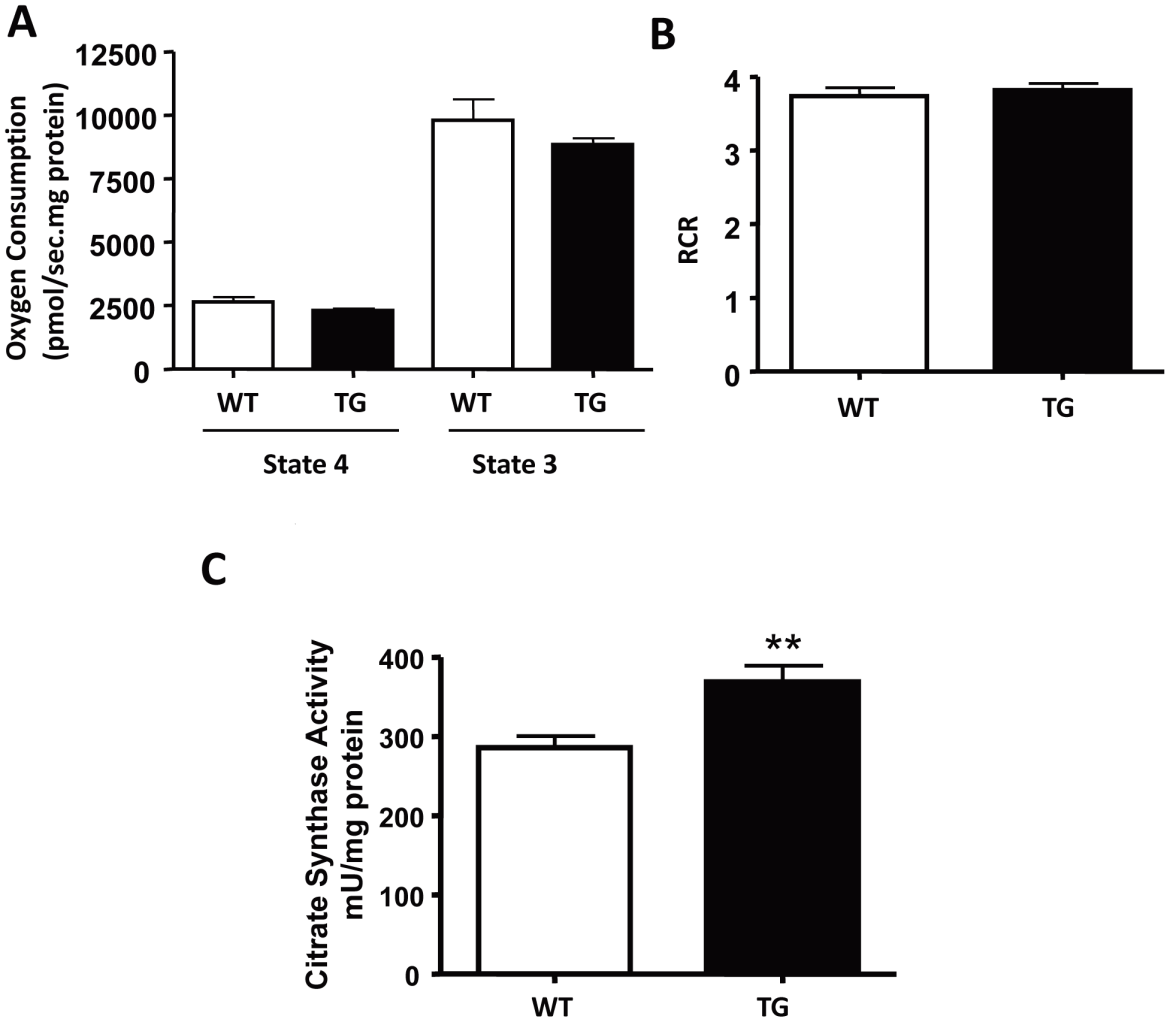
Mitochondrial respiration rates and citrate synthase activity in muscles of MCK-SIRT3M3 transgenic mice. (A): Oxygen consumption rates in isolated mitochondria from muscles of WT and MCK-SIRT3M3 mice at 5 months of age (n = 6). Respiration parameters were recorded using an Oroboros O2k oxygraph. Resting respiration (state 4) and maximal ADP-stimulated respiration (state 3) were presented. (B): Respiratory control ratio (RCR) was calculated as the ratio of oxygen consumption at state 3 over oxygen consumption at state 4. (C): Citrate synthase (CS) activity in gastrocnemius muscle extracts from WT and MCK-SIRT3 transgenic mice at 5 months of age (n = 6). CS activity was measured according to Srere [71]. **P<0.01 between WT and MCK-SIRT3M3 mice.

When we isolated mitochondria from gastrocnemius muscle and measured mitochondrial respiration, we found no difference in mitochondrial oxygen consumption (Fig. 6A) or respiratory control ratio (Fig. 6B) between transgenic mice and WT controls. However, we found a significant increase of citrate synthase levels in the muscle of the transgenic mice (Fig. 6C). The amount of mitochondria proteins (mg) isolated from per gram of muscle also increased (WT: 2.65 mg/g, TG: 3.42 mg/g; p = 0.06). These results suggest that although there is no change in mitochondrial respiration, mitochondrial density increases in the muscle of the transgenic mice.

### Transgenic Expression of SIRT3M3 caused Muscle Atrophy through Up-regulation of FOXO1

**Figure 7:**
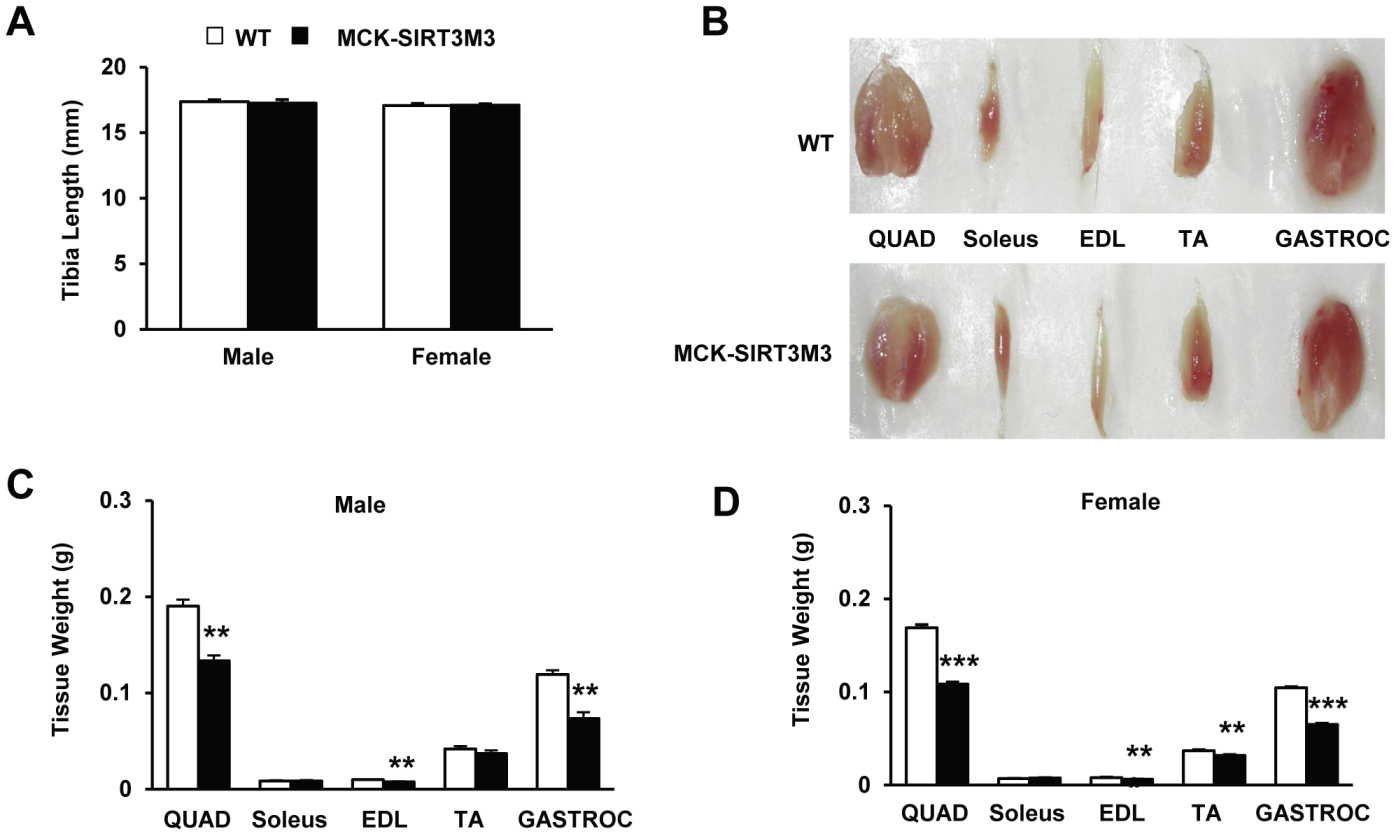
Transgenic expression of SIRT3M3 decreased skeletal muscle mass. (A): Tibia length of WT and MCK-SIRT3M3 mice. (B): Comparison of representative samples of dissected skeletal muscle (Quad, quadriceps; EDL, extensor digitorum longus; TA, tibialis anterior; Gastroc, gastrocnemius) between MCK-SIRT3M3 mice and litter-mate control mice. (C and D): Muscle weights from 6–8 m old WT and MCK-SIRT3M3 mice, for male and female. *n* = 6–8. **P<0.01, ***P<0.001 between WT and MCK-SIRT3M3 mice.

**Figure 8:**
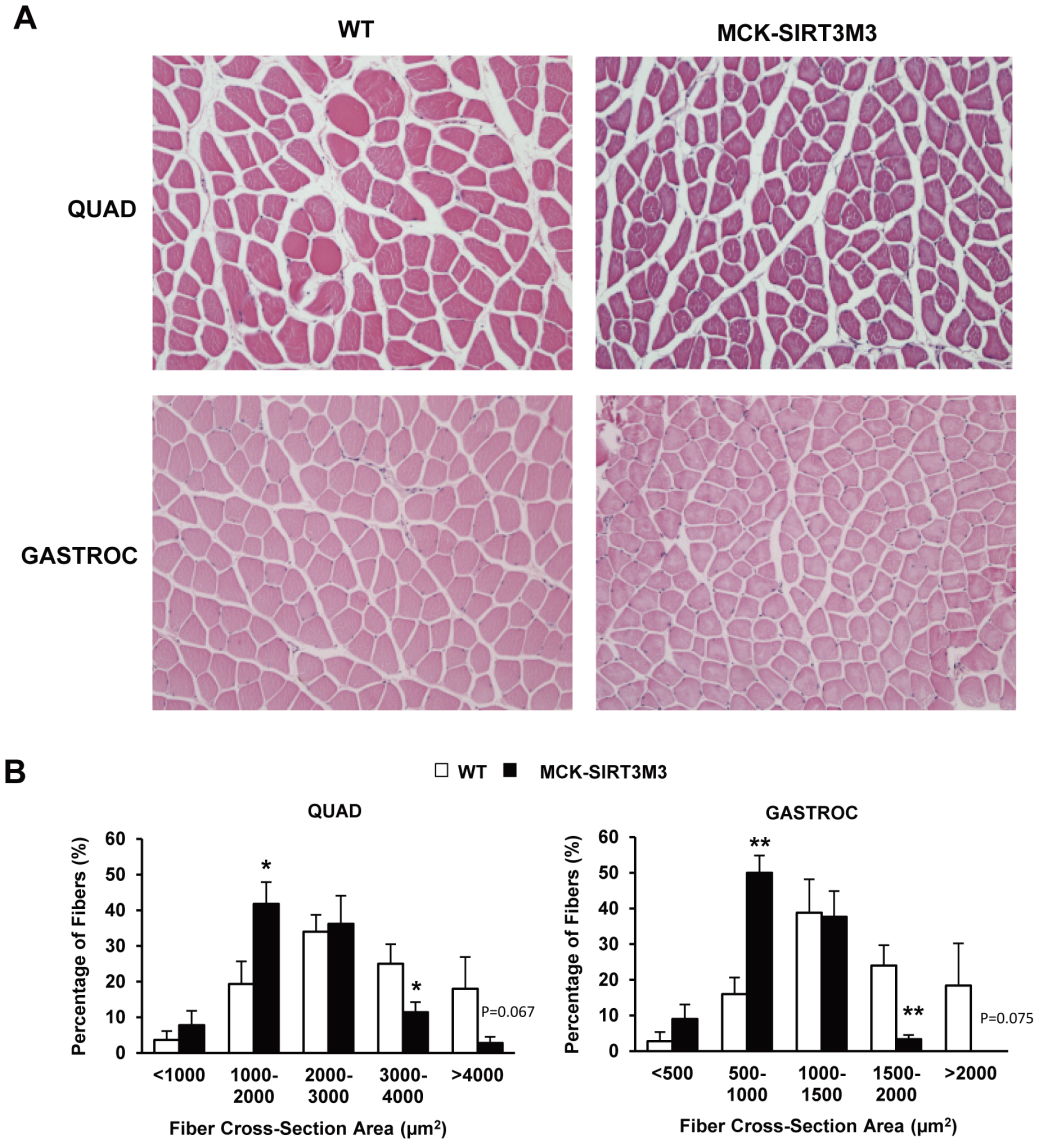
Fiber size of muscle from MCK-SIRT3M3 transgenic mice. (A): H&E staining of quadriceps and gastrocnemius muscle from 3–4 month-old WT and MCK-SIRT3M3 mice. (B): Fiber cross-section area of quadriceps and gastrocnemius muscle from 4.5 m old male WT and MCK-SIRT3M3 mice. *n* = 3–5. *P<0.05, **P<0.01 between WT and MCK-SIRT3M3 mice.

We found that tibia lengths of both male and female mice showed no change, indicating no difference of linear growth between WT and transgenic mice (Fig. 7A and S4A). However, we found that MCK-SIRT3M3 mice have significantly smaller muscles (Fig. 7B). The quadriceps, extensor digitorum longus (EDL), tibialis anterior (TA), and gastrocnemius muscles from MCK-SIRT3M3 mice (bottom row) were apparently smaller than those from the WT mice (top row). The MCK-SIRT3M3 mice have lower muscle weight than that of the control mice, especially in the quadriceps, EDL, and gastrocnemius (Fig. 7C, 7D, S4B and S4C). The weight of soleus muscle was not affected, which might be due to the already high expression of endogenous SIRT3 in this muscle. In contrast, the weights of other tissues, such as heart, liver, and white and brown adipose tissues, were unchanged in MCK-SIRT3M3 mice (data not shown). Furthermore, H&E staining revealed that quadriceps muscle and gastrocnemius muscle in the transgenic animals have smaller fibers (Fig. 8A and S5). Measurement of fiber cross-section area revealed that quadriceps and gastrocnemius from MCK-SIRT3M3 mice have significantly more small fibers but less large fibers (Fig. 8B). Thus, expression of SIRT3 in skeletal muscle causes a reduction of muscle mass, which contributes to the reduction of the lean body mass.

**Figure 9:**
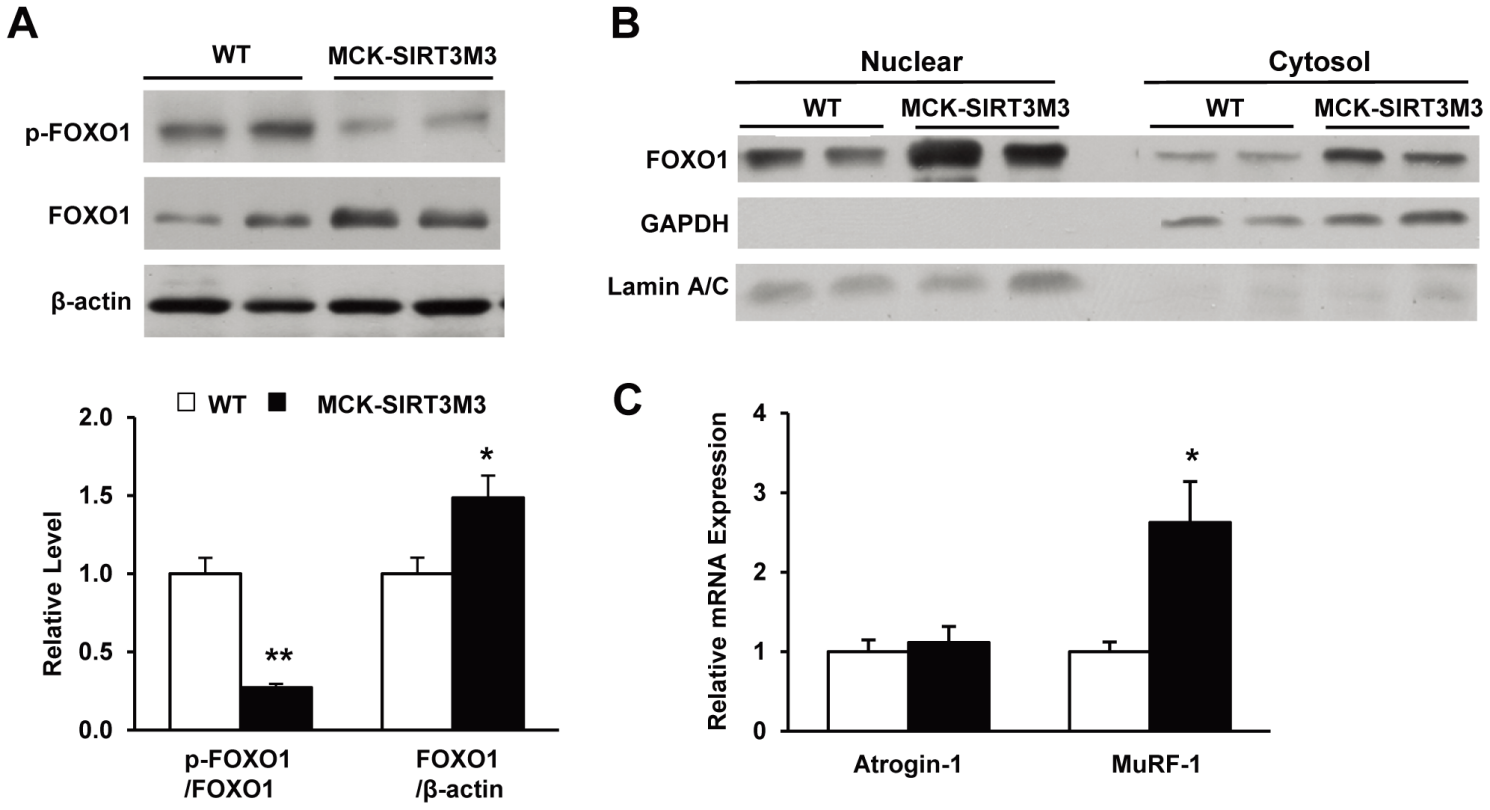
MCK-SIRT3M3 mice had increased muscle FOXO1 expression. (A): Total and phosphorylated FOXO1 protein level in quadriceps muscle from WT and MCK-SIRT3M3 mice. (B): Total FOXO1 protein level in nuclear and cytosol fraction of quadriceps muscle from WT and MCK-SIRT3M3 mice. (C): q-PCR analyses of MuRF-1 and atrogen-1 in quadriceps muscle from WT and MCK-SIRT3M3 mice. *n* = 6. *P<0.05 between WT and MCK-SIRT3M3 mice.

FOXO1 is a key mediator of muscle protein degradation. Overexpression of FOXO1 in skeletal muscle can cause muscle atrophy [36], [37]. The total FOXO1 protein level was significantly increased in the muscle of MCK-SIRT3M3 mice, whereas the phosphorylated FOXO1 level was decreased (Fig. 9A). Further analysis revealed that the FOXO1 protein level was elevated in both nuclear and cytosol of muscle of transgenic mice muscle (Fig. 9B). Because phosphorylation of FOXO1 negatively regulates FOXO1 activity, an increase of the FOXO1 protein level and a decrease of the FOXO1 phosphorylation should enhance the FOXO1 action. As FOXO1 activates the expression of the E3 ubiquitin ligases, atrogin-1/MAFbx and MuRF-1, which participate in muscle atrophy [35], we then detected the transcriptional levels of atrogin-1 and Murf-1. We found the mRNA level of Murf-1 but not atrogin-1 was increased in the muscle of MCK-SIRT3M3 mice (Fig. 9C). Thus, our data indicate that MCK-SIRT3M3 mice are likely to have increased muscle protein breakdown through an up-regulation of FOXO1 activity and the expression of MuRF-1.

## Discussion

To gain insight into the role of SIRT3 in skeletal muscle *in vivo*, we generated SIRT3 transgenic mice. The SIRT3M3 transgene was predominantly expressed in the skeletal muscle and partly in heart tissue, but not in other tissues. We have established multiple lines of the transgenic mice. The shared phenotypes of two independent transgenic lines might assure that the phenotype we have observed are not due to a positional effect. In addition, our observation of increased muscle AMPK activation and oxidative capacity of the transgenic mice is consistent with previous findings of down-regulation of muscle AMPK phosphorylation and oxygen consumption in SIRT3 knockout mice [7], [24]. These results suggest that the phenotype of the SIRT3M3 transgenic mice is caused by the transgene expression.

The calorimetry study showed that MCK-SIRT3M3 mice had lower RER. The lower RER indicates a preference for lipids usage as a fuel source for these mice. This finding is in agreement with SIRT3’s role in promoting fatty acid oxidation [10]. This is also consistent with muscle fiber type switch and the activation of AMPK and PPARδ. Skeletal muscle possesses four fiber types, I, IIa, IIx, and IIb, in the order of decreasing oxidative capacity and increasing glycolytic preference [60]. Type I muscle fibers have slow-twitch contraction characteristics, high mitochondrial content, and fatigue resistance. Type I fibers also have higher rates of glucose and fatty acid uptake and greater oxidative capacity [61]. Interestingly, we found that the number of type I fibers was significantly increased in the skeletal muscle of MCK-SIRT3M3 mice. These results suggest that SIRT3 is a positive regulator of fiber type switch towards type I fiber. Consistently, the MCK-SIRT3M3 mice showed increased exercise performance but lower muscle strength. The altered distribution of fiber types is also likely to contribute to the increased utilization of lipids as a fuel source. We found that AMPK was dramatically activated in the skeletal muscle of MCK-SIRT3M3 mice. Additionally, PPARδ protein level was also up-regulated in skeletal muscle of MCK-SIRT3M3 mice. Both AMPK and PPARδ promote type I fiber formation and mitochondrial biogenesis [48], [62]. We found that transgenic expression of SIRT3 increases muscle mitochondrial density. However, because the transgenic mice have smaller muscles, the total number of mitochondria per muscle is not changed. Taken together, SIRT3 could activate AMPK and PPARδ, to regulate the fiber switch. At this moment, how SIRT3M3 activates AMPK and PPARδ is not clear. Researchers have reported that SIRT3 deacetylates and activates LKB1, an upstream kinase of AMPK [63]. Therefore, it is possible that SIRT3 activates AMPK through LKB1.

The MCK-SIRT3M3 mice exhibited significant decreases of muscle mass. The weight of skeletal muscles, such as quadriceps and gastrocnemius, decreased more than 30%, whereas the tibia length did not change. It is conceivable that nutrient deprivation, such as caloric restriction or fasting, results in the breakdown of muscle proteins to mobilize amino acids for the use of other tissues, such as liver for glucose production [5]. The activation of SIRT3 in muscle during nutrient deprivation might mediate this process [8]. We found the total FOXO1 protein level was increased and the phosphorylated FOXO1 level was decreased in muscle of the SIRT3 transgenic mice. The expression of one of the FOXO-targeted atrogene, MuRF-1, was also up-regulated. This offers one mechanistic explanation for muscle atrophy. AMPK might also contribute to muscle atrophy. Muscle AMPK is activated by denervation [64]. The AMPK activator, AICAR, elevates the expression of FOXO1 and FOXO3 in mouse muscle [65] but inhibits mTOR activation. Although AICAR activates IGF-1-stimulated Akt activation, it decreases FOXO3 phosphorylation to increase FOXO3 nuclear localization and the expression of atrogin-1 and MuRF-1 in C2C12 cells [66]–[68]. Possibly, AMPK directly phosphorylates FOXO1 to suppress FOXO1 degradation [69] and increases FOXO1 transactivation of MuRF-1 [70].

Collectively, our data provide strong evidence that induction of SIRT3 expression in skeletal muscle promotes switching of muscle fiber types, improves muscle oxidative capacity, and changes muscle mass.

## Supporting Information

Figure S1 **Body weight and metabolic characterization of the second line of MCK-SIRT3M3 transgenic mice.** (A): Body weight of male WT and MCK-SIRT3M3 mice. *n* = 6–9. (B): Body weight of female WT and MCK-SIRT3M3 mice. *n* = 5–9. (C): Daily food intake of 8-month old WT and MCK-SIRT3M3 mice. (D): Total locomotor activity at daytime and nighttime of 8-month old WT and MCK-SIRT3M3 mice. (E): Respiratory exchange rate (RER) of WT and MCK-SIRT3M3 mice. *n* = 6. *P<0.05 between WT and MCK-SIRT3M3 mice.
(TIF)

Figure S2 **Glucose tolerance tests (GTT) of MCK-SIRT3M3 transgenic mice.** The GTT of WT and MCK-SIRT3M3 mice at 3–5 months of age. (A): The first transgenic line male mice; (B): The first transgenic line female mice; (C): The second transgenic line male mice; (D): The second transgenic line female mice. *n* = 6–9.
(TIF)

Figure S3 **Muscle performance of the second line of MCK-SIRT3M3 transgenic mice.** (A): Running distance of WT and MCK-SIRT3M3 mice on treadmill. (B): Holding time of WT and MCK-SIRT3M3 mice on grid mesh test. (C): Transition time of WT and MCK-SIRT3M3 mice climbing on string test. *n* = 6–10. *P<0.05, **P<0.01 between WT and MCK-SIRT3M3 mice.
(TIF)

Figure S4 **Tibia length and muscle weight of the second line of MCK-SIRT3M3 transgenic mice.** (A): Tibia length of WT and MCK-SIRT3M3 mice. (B and C): Muscle weights from 6–8 m old WT and MCK-SIRT3M3 mice, for male and female. QUAD, quadriceps; EDL, extensor digitorum longus; TA, tibialis anterior; GASTROC, gastrocnemius. *n* = 6–7. *P<0.05, **P<0.01 between WT and MCK-SIRT3M3 mice.
(TIF)

Figure S5 **Lower magnification (5X) images of H&E staining of quadriceps and gastrocnemius sections from 3–4 month-old WT and MCK-SIRT3M3 mice.**
(TIF)
