# Supplementary figures and images for: Regulation of Skeletal Muscle Oxidative Capacity and Muscle Mass by SIRT3
Source: PLoS One. 2014 Jan 15;9(1):e85636. doi: 10.1371/journal.pone.0085636 (PMC3893254; doi:10.1371/journal.pone.0085636)

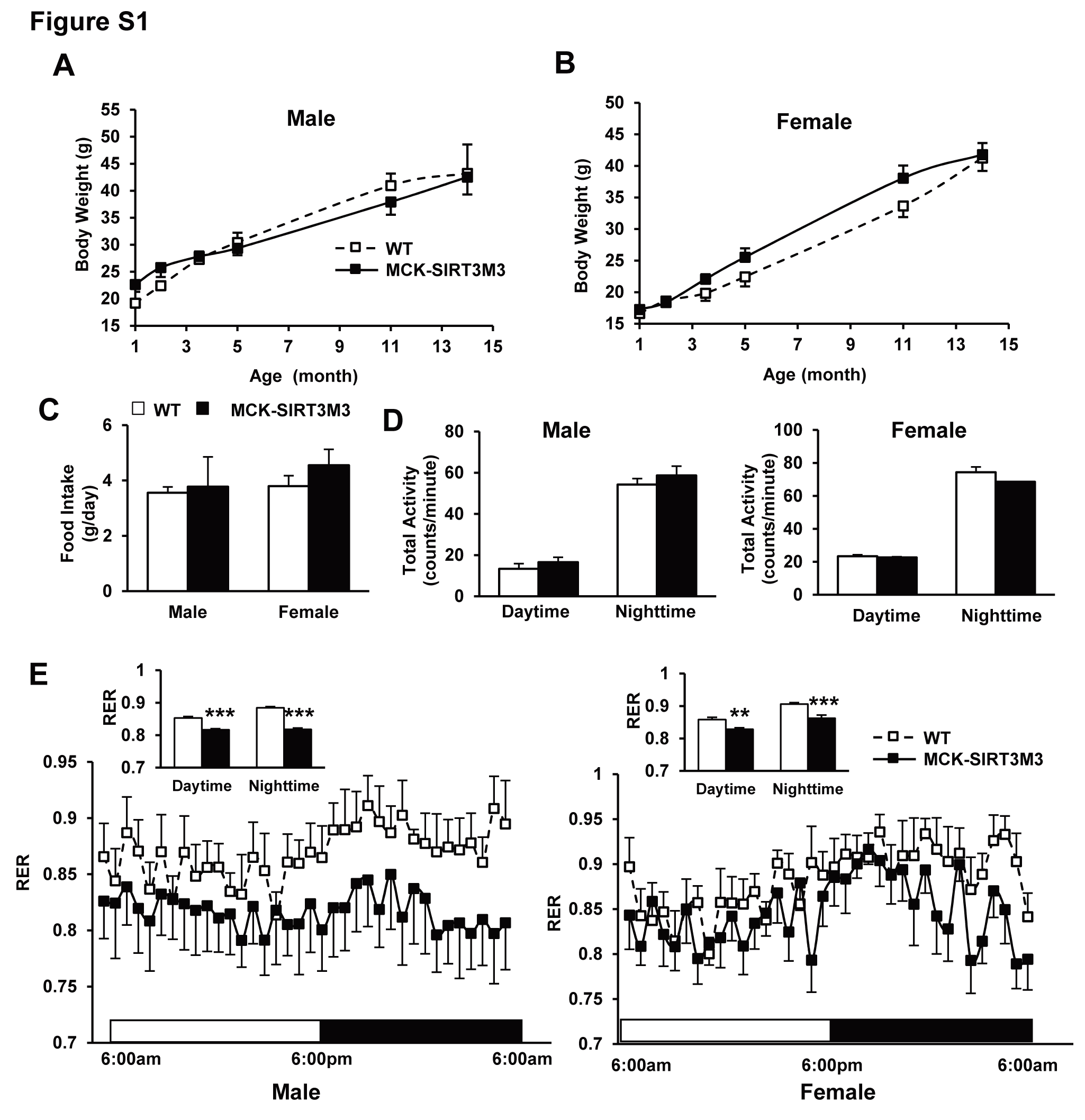

Supplement: Figure S1 — Body weight and metabolic characterization of the second line of MCK-SIRT3M3 transgenic mice. (A): Body weight of male WT and MCK-SIRT3M3 mice. n = 6–9. (B): Body weight of female WT and MCK-SIRT3M3 mice. n = 5–9. (C): Daily food intake of 8-month old WT and MCK-SIRT3M3 mice. (D): Total locomotor activity at daytime and nighttime of 8-month old WT and MCK-SIRT3M3 mice. (E): Respiratory exchange rate (RER) of WT and MCK-SIRT3M3 mice. n = 6. *P<0.05 between WT and MCK-SIRT3M3 mice. (TIF) [file pone.0085636.s001.tif]

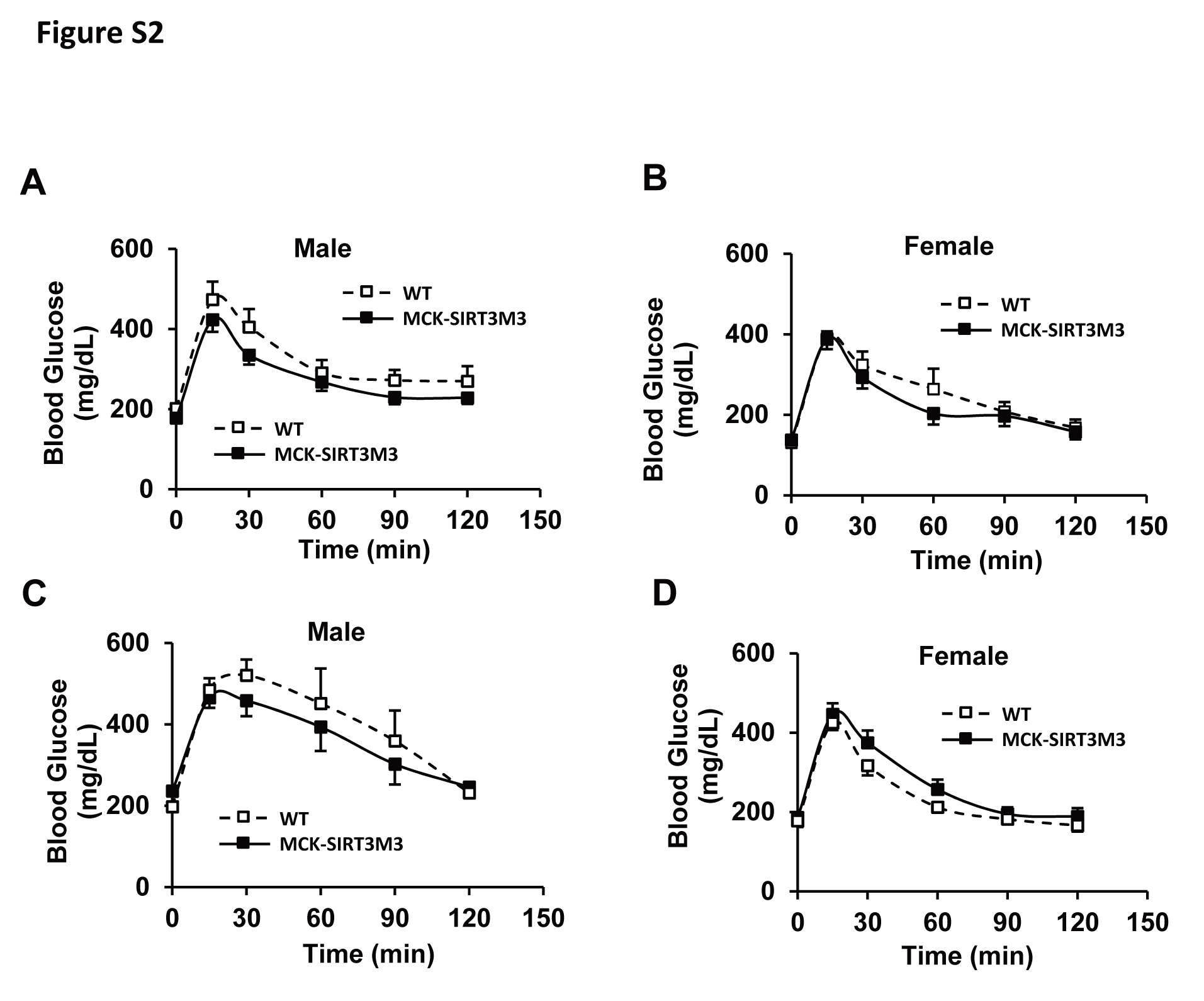

Supplement: Figure S2 — Glucose tolerance tests (GTT) of MCK-SIRT3M3 transgenic mice. The GTT of WT and MCK-SIRT3M3 mice at 3–5 months of age. (A): The first transgenic line male mice; (B): The first transgenic line female mice; (C): The second transgenic line male mice; (D): The second transgenic line female mice. n = 6–9. (TIF) [file pone.0085636.s002.tif]

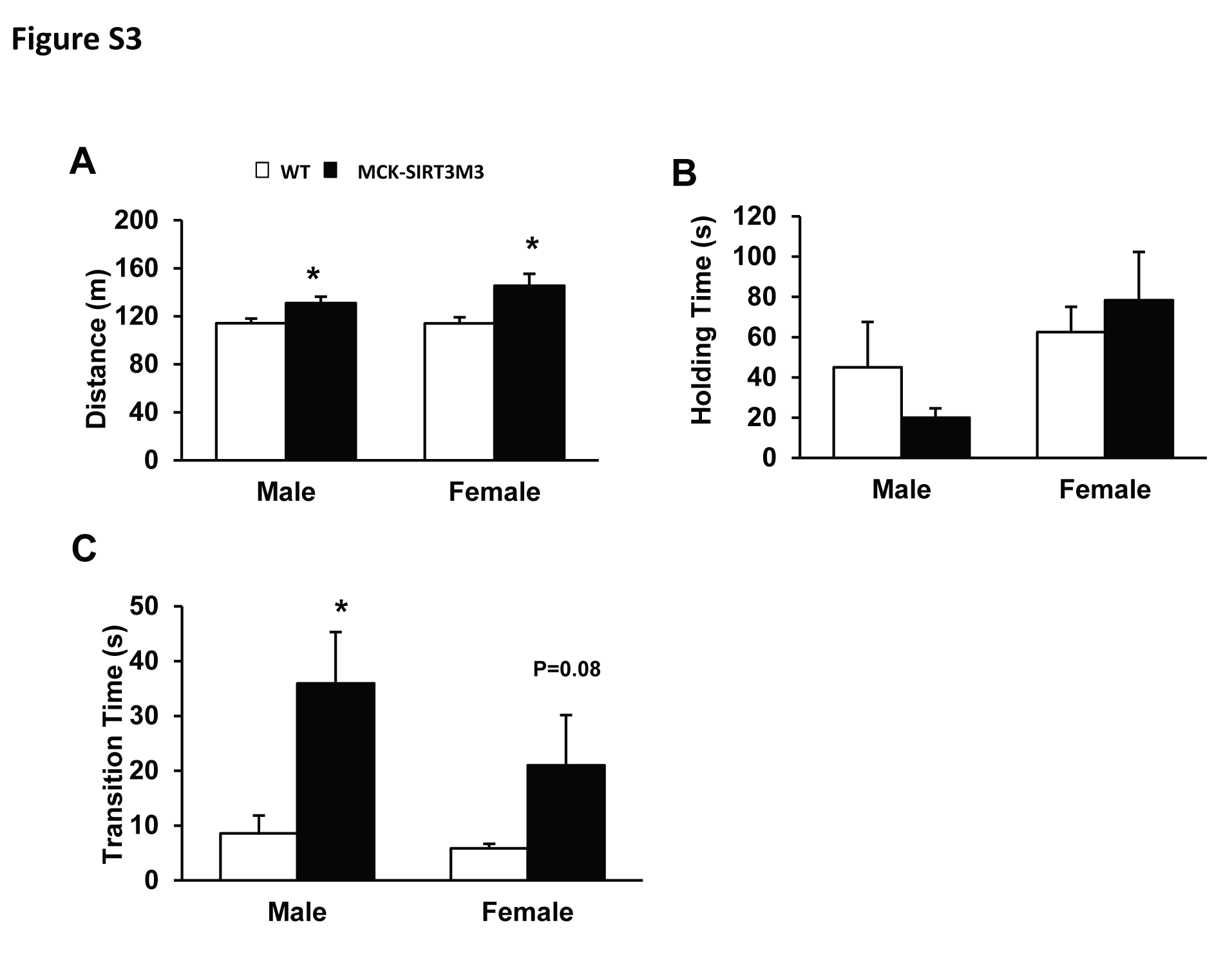

Supplement: Figure S3 — Muscle performance of the second line of MCK-SIRT3M3 transgenic mice. (A): Running distance of WT and MCK-SIRT3M3 mice on treadmill. (B): Holding time of WT and MCK-SIRT3M3 mice on grid mesh test. (C): Transition time of WT and MCK-SIRT3M3 mice climbing on string test. n = 6–10. *P<0.05, **P<0.01 between WT and MCK-SIRT3M3 mice. (TIF) [file pone.0085636.s003.tif]

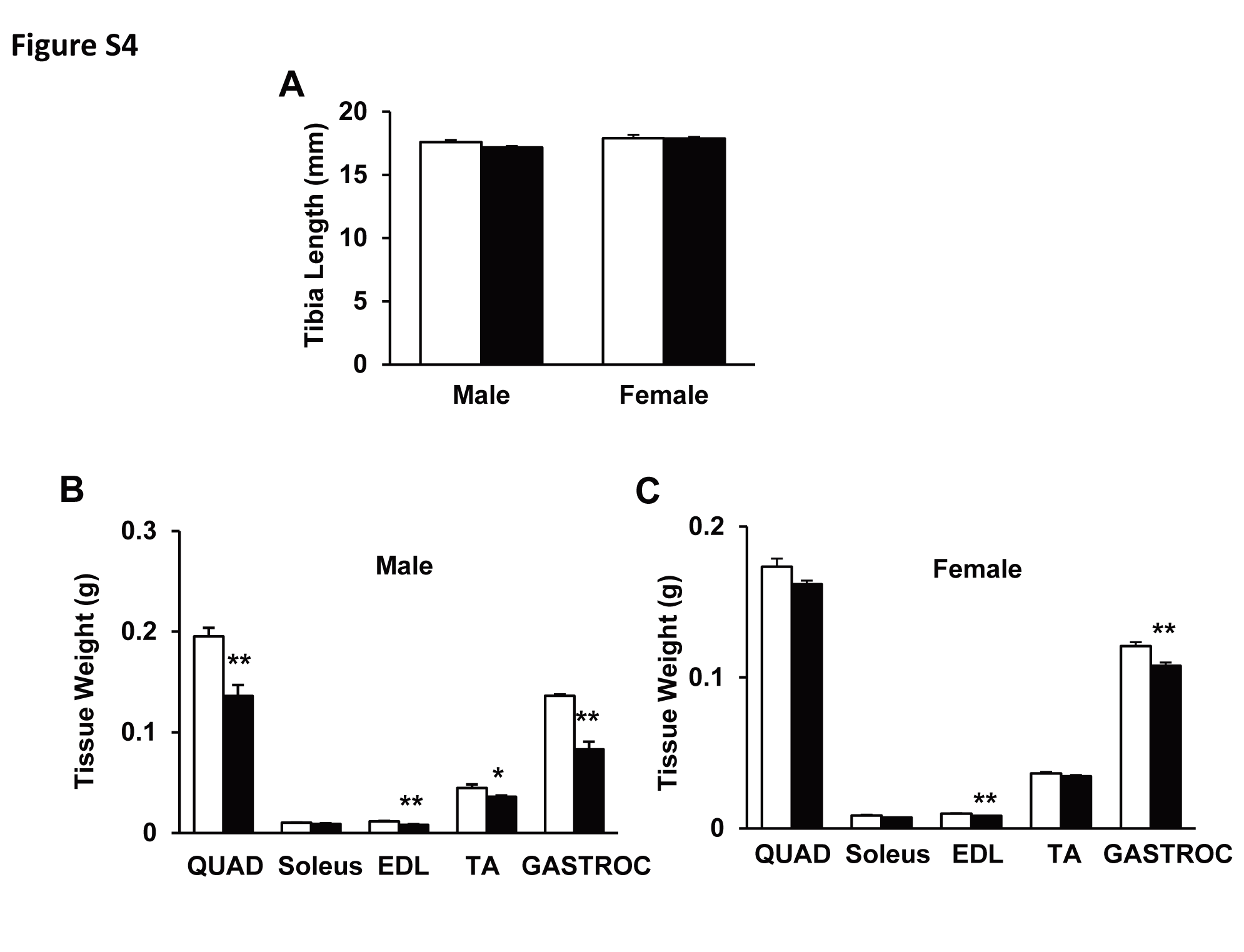

Supplement: Figure S4 — Tibia length and muscle weight of the second line of MCK-SIRT3M3 transgenic mice. (A): Tibia length of WT and MCK-SIRT3M3 mice. (B and C): Muscle weights from 6–8 m old WT and MCK-SIRT3M3 mice, for male and female. QUAD, quadriceps; EDL, extensor digitorum longus; TA, tibialis anterior; GASTROC, gastrocnemius. n = 6–7. *P<0.05, **P<0.01 between WT and MCK-SIRT3M3 mice. (TIF) [file pone.0085636.s004.tif]

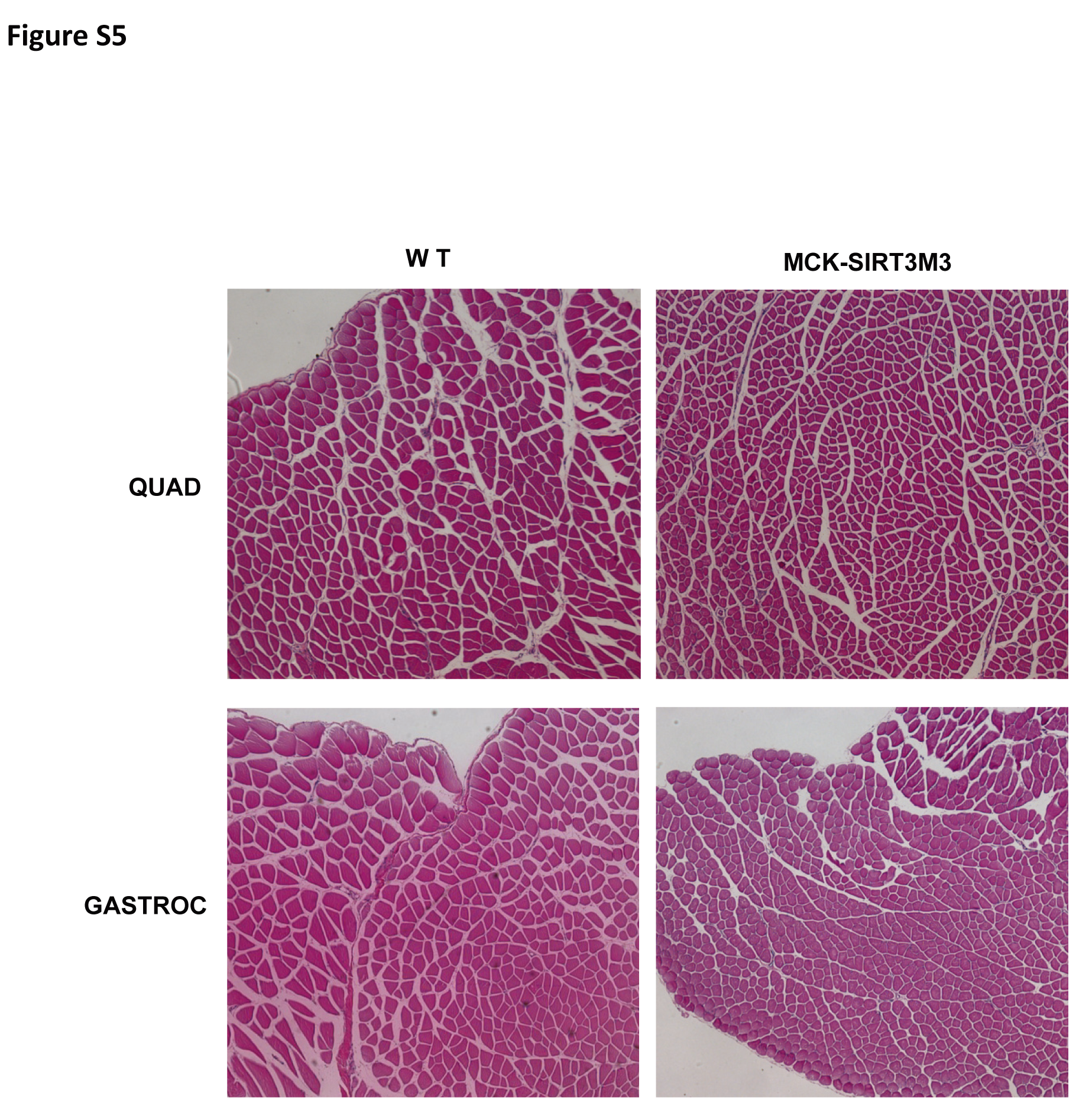

Supplement: Figure S5 — Lower magnification (5X) images of H&E staining of quadriceps and gastrocnemius sections from 3–4 month-old WT and MCK-SIRT3M3 mice. (TIF) [file pone.0085636.s005.tif]
